# Mechanisms and histopathological impacts of acetamiprid and azoxystrobin in male rats

**DOI:** 10.1007/s11356-021-18331-3

**Published:** 2022-01-29

**Authors:** Heba Nageh Gad EL-Hak, Rasha A. Al-Eisa, Lamia Ryad, Ekramy Halawa, Nahla S. El-Shenawy

**Affiliations:** 1 Zoology Department, Faculty of Science, Suez Canal University, Ismailia, 41522 Egypt; 2 Department of Biology, College of Sciences, Taif University, P.O. Box 11099, Taif, 21944 Saudi Arabia; 3 Agricultural Research Center, Central Lab of Residue Analysis of Pesticides and Heavy Metals in Food, Giza, Egypt

**Keywords:** Lutilizing hormone, Follicle-stimulating hormone, Nitric oxide, Calcium, Sperm count, Male rat

## Abstract

Acetamiprid (neonicotinoid insecticide) and azoxystrobin (fungicide) are widespread pesticides used for pest management, but they have the potential for toxicity to mammals. The goal of this study was to look for oxidative stress, metabolic alterations, and reproductive problems in male rats’ serum after 2 months of exposure to sub-lethal dosages of acetamiprid and azoxystrobin. Seven classes of male rats were formed: control, 3 groups of acetamiprid (1/10, 1/20, 1/40 LD_50_), and 3 groups of azoxystrobin (1/10, 1/20, 1/40 LD_50_) and were orally daily treated (*n* = 8/group). Our findings revealed that acetamiprid and azoxystrobin disrupted oxidative and metabolic processes in the examined rats throughout 30 and 60 days of testing. The levels of nitric oxide increased significantly, while catalase, a superoxide dismutase enzyme, and glutathione reductase activity were reduced. Serum levels of sex hormones, calcium, and total protein have all dropped substantially in rats. In comparison to the control group, the testis and liver structure, as well as spermatozoa parameters, had distinct histological characteristics. In conclusion, acetamiprid and azoxystrobin exhibit dose- and time-dependent effects on oxidative parameters that cause testis damage.

## Introduction

Free radicals called reactive oxygen species (ROS) can infiltrate DNA, causing a unique sequence of DNA changes. The bulk of ROS is made up of the superoxide anion, H_2_O_2_, and singlet oxygen (Butterfield 2020). ROS are considered to be caused mostly by endocrine-disrupting chemicals (EDCs). One of the EDCs was pesticides (Chang et al. 2009; Pouech et al. 2012). Acetamiprid (AC), a neonicotinoid pesticide, binds to postsynaptic nicotinic acetylcholine receptors in insects’ central nervous systems (Pohanish 2015). Azoxystrobin (AZ) is a fungal disease inhibitor used in agriculture (EPA 1997). It acts by blocking electron transport in the mitochondria. There is evidence that ROS has a role in various diseases as a result of inducing oxidative stress (Halliwell 2006). In fruit and vegetable samples, AZ residues equivalent to or below maximum residue levels (MRLs) were found. Values for AZ bioaccumulation potential or biomagnifications have been scarce (Rodrigues et al. 2013). Also, marked immunosuppressive effects were observed for AZ (Naasri et al. 2021).

Antioxidants, including enzymatic and non-enzymatic defense mechanisms, defend against ROS damage. The enzymes’ superoxide dismutase (SOD), glutathione (GSH), and catalase (CAT) (Halliwell 2006; Bond and Greenfield 2007), as well as glutathione reductase (GR), are all part of the enzymatic defense system. CAT is an antioxidant enzyme that catalyzes the reduction of H_2_O_2_ to water and can dissolve organic hydroperoxides. CAT uses H_2_O_2_ to oxidize toxins such as phenols, formic acid, formaldehyde, and alcohol (Nazıroğlu 2007). ROS has been shown to damage several ion channels, including calcium channels (Dringen 2005). Excess intracellular Ca^2+^ levels may thereby trigger CAT breakdown, impair normal mitochondrial function, and result in oxidative stress and bioenergetic failure (Nilius et al. 2008).

Total protein, albumin, and globulin levels in blood plasma are essential components that assist to maintain osmotic pressure and transporting steroid hormones, lipids, and fibrinogen in blood clotting. Edema is characterized by the movement of fluid from blood arteries into tissues, which is caused by low albumin levels. A lower-than-normal albumin/globulin ratio can suggest problems with the kidneys and hepatocytes (Riris 2017). The presence of phthalate esters on Leydig cells reduced protein levels dramatically (Di Lorenzo et al. 2020). Furthermore, female rats exposed to biphenyl amine had lower blood total protein levels and altered antioxidant enzyme levels (Moustafa and Ahmed 2016). Moreover, dichlorvos causes oxidative stress by releasing ROS (Kanu et al., 2016).

Oxidative stress occurs when the production of harmful chemicals known as free radicals outpaces the antioxidant defenses’ ability to protect (Alia et al. 2011). Antioxidant activity is disturbed, indicating a shift in blood cell oxidative state. Following earlier observations (Kapoor et al. 2011), SOD, CAT, and GSH levels increased.

In several species, the AC has been proven to be harmful to reproduction (Kenfack et al. 2018; Terayama et al. 2018). Arcan et al. (2020) studied the reproductive toxicity of AC in male rats given 12.5 mg/kg, 25 mg/kg, or 35 mg/kg orally for 90 days. Sperm count and plasma testosterone levels both dropped in a dose-dependent manner, according to their findings. GnRH, FSH, and LH levels increased at low and medium dosages, whereas AC induced lipid peroxidation (LPO) and glutathione (GSH) to diminish in the testes. AC increased LPO and nitric oxide (NO) levels of Leydig cells.

In Leydig cells, AC was also discovered to reduce the production of adenosine triphosphate (ATP) and cyclic adenosine monophosphate (cAMP) (Ibrahim et al. 2020). Because A signaling routes are the major signaling mechanisms of steroidogenesis, ROS generated by AC can block the signal pathway, resulting in decreased testosterone production. It reduced the activities of CAT, glutathione peroxidase (GP), and superoxide dismutase (SOD) in mice testes, resulting in excessively high ROS levels (Ibrahim et al. 2020).

According to our knowledge, there is no data available on the toxicity of AC and AZ on the hormones and their histopathological effects on the liver and testis. Therefore, the study was concerned with the evaluation of male reproductive toxicity of AC and AZ and their mechanisms to confirm whether these compounds can act as EDCs or not.

## Material and methods

### Chemical and reagent

Acetamiprid and azoxystrobin used for the experiment were obtained from Dr. Ehrenstorfer GmbH (Augsburg, Germany, CHEM Europe. De-ionized water (DIW) was available through a Millipore water purification system.

### Experimental design and sample collection

The experiments were conducted following the European Directive 2010–63-EU (2010) and approved by the Local Ethics Committee at Suez Canal University, Faculty of Science (NO. REC57/2021). Rats were provided by the National Research Centre’s (NRC) Animal Breeding House, Dokki, Giza, Egypt. All efforts were made to minimize the suffering and the number of animals used. Fifty-six adult male Wister rats weighing 140–160 g at age 60–80 days were used. They were kept together under observation for two weeks before the beginning of the experiment for acclimation under standardized conditions. The rats were housed in metal cages at a 24 ± 3 °C temperature with normal light conditions (12-h light/dark cycle) and received food and water according to ad libitum feeding practice (Ritskes-Hoitinga et al. 2012).

For dosing reasons, individual standard solutions of 50 mg/mL AZ and 3000 g/mL AC were prepared weekly in water. The LD_50_ of AC was calculated to be 200 mg/kg (EC 2004; EFSA 2010; Williams 2013), while the EPA estimated the LD_50_ of azoxystrobin to be 5000 mg/kg (EPA 1997). After 2 weeks of acclimatization, the animals were divided into seven groups of eight rats, with group 1 serving as the control group. Oral gavage was used daily to provide (1/10, 1/20, and 1/40) LD_50_ of AC (20, 10, and 5 mg/kg b wt., respectively) to groups 2, 3, and 4. The LD_50_ of AZ was provided to groups 5, 6, and 7 (1/10, 1/20, and 1/40, respectively) (500, 250, and 125 mg/kg b wt.).

Blood samples were taken either after a month or 2 months. The mix of ketamine and xylazine at a dose of 80–100 mg/kg and 5–10 mg/kg, respectively, was used as an anesthetic agent and injected intraperitoneal. Blood was collected from the rat’s retro-orbital using capillary tubes. Whole blood was placed in an empty tube, allowed to clot, and then centrifuged at 500 xg for 15 min to obtain serum. For biochemical analysis, the serum was maintained at − 80 °C.

### Biomarker determination

The measurement of serum calcium is based on the formation of a color complex (colorimetric technique) between calcium and o-cresolphtalein in an alkaline medium. The color strength in the sample is proportional to the calcium concentration (Ng 2002). At a wavelength of 570 nm, the color was measured at a temperature of 37 °C and the value was presented as mg/dL.

In an alkaline medium, proteins form an intense violet-blue complex with copper salts. As an antioxidant, iodide is used. The color strength in the assay is proportional to the overall total protein (TP) content (Eckfeldt 1999). The value was calculated using a wavelength of 570 nm and a temperature of 37 °C, and it was expressed as mg/dL.

Within 15 min after applying the stop solution, the optical density was read at 450 nm using an ELISA reader to determine the SOD (Okwakpam and Monanu 2020). The CAT was determined using ELISA (Chelikani et al. 2004).

The biotin-conjugated antibody and enzyme-conjugated Avidin were used to determine GR. The color transition is calculated spectrophotometrically at a wavelength of 450 nm after the enzyme–substrate reaction is terminated by adding a sulphuric acid solution (Elshal and McCoy 2006).

The detecting antibody was a polyclonal antibody with biotin labeled ELISA Kit, and the pre-coated antibody was Rat NO monoclonal antibody. The color depth of the samples and the testing parameters were highly linked (Report 2014).

### Hormonal evaluation

In this experiment, the competitive inhibition enzyme immunoassay method is employed. On the microtiter plate included in this kit, the goat-anti-rabbit antibody has been pre-coated. Using an antibody specific for luteinizing hormone (LH) and LH conjugated with Horseradish Peroxidase, standards or samples are added to the appropriate microtiter plate wells (HRP). The antibody is used to initiate a competitive inhibitory response between HRP-labeled LH and LH that has not been tagged. Using an ELISA reader, the optical density (OD) was measured at 450 nm (Report 2014).

The goat-anti-rabbit antibody has been pre-coated on the microtiter plate used in this kit. The antibody is used to start a competitive inhibitory response between FSH that has been HRP labeled and FSH that has not been tagged. Using an ELISA reader, the optical density (OD) was measured at 450 nm (Report 2014).

### Sperm count and mortality determination

The caudal epididymis was quickly removed from anesthetized rats. The adherent fat, blood vessels, and connective tissue were cut away and the organ from each animal was placed on a hollow plate. The sperm was released by cutting the cauda epididymis longitudinally with a pair of fine-pointed scissors and compressed with forceps and was therefore deposited free of epididymal tissue into the cavity (Adamkovicova et al. 2016).

The sperm suspension was drawn into a white blood cell pipette and diluted to 1:100 with proteolytic enzyme solutions with collagenase (No. C-2139, SIGMA S 1. Louis, MO, USA) or trypsin (DIFCO Detroit, MI, USA) in Ringer’s phosphate solution. After this procedure, a final 1:1000 dilution was performed with formaldehyde saline fixative (1.8% NaCI and 2.0% formalin).

A hemocytometer (BOP NO. 2936-FIO, Arthur H. Thomas Co., Philadelphia, PA, USA) with improved double Neubauer ruling was used for the counting of spermatozoa. Counts for 2–4 hemocytometer chambers were averaged (Adamkovicova et al. 2016)**.** Motion parameters include the percentage of motile spermatozoa (MOT) that was determined, as well as the percentage of mortality.

### Histopathological evaluation

The 10% formalin was used to fix tissue samples from the liver and testes. Fixed tissues were dehydrated for 30 min in ascending series of alcohol (70, 80, 90, 95, and 100%) with gentle shaking before being immersed in absolute ethanol overnight. The tissues were soaked in xylene (3 times) for 30 min before being paraffin at 56 °C (30 min, 3 times). The tissues were embedded in paraffin and cut into 4-μm sections using a digital semi-automatic microtome, microtome Ambala (Haryana, India). The samples were mounted on microscope slides, which were then stained for 5 min with hematoxylin and eosin (Bancroft and Gamble 2013). The stained slides were examined and photographed by using an Axiostar Plus (Carl Zeiss, Göttingen, Germany) microscope adapted with Canon (Pc 1200 Power shoot A641) digital camera using Zoom Browser Ex software at the central lab of Zoology Department, Faculty of Science, Suez Canal University.

### Statistical analysis

The mean and standard error are used to analyze the data. The significance level was set at *P* ≤ 0.05 using two-way ANOVA followed by Duncan’s multiple range test to estimate the particular difference between pairs of the mean.

## Results

The effects of sub-lethal dosages of AC and AZ were assessed by measuring calcium, proteins, antioxidant enzymes (SOD, CAT, and GR), as well as the NO levels in the rats’ serum.

### Biochemical evaluation

Table 1 shows that AC and AZ caused a noticeable difference at high doses in rat serum calcium (Ca) concentration as compared to normal rats. There was a slight and moderate difference induced by low doses. The AC and AZ, after 2 months of treatment with 5 and 125 mg/kg/day, respectively, Ca levels were 11.09 mg/dL and 12.20 mg/dL, respectively. However, at 20 and 500 mg/kg/day of AC and AZ, Ca levels were decreased to be 8.54 and 10.36 mg/dL, respectively, with a Ca concentration of 13.29 mg/dL for the control group.

**Table 1:** Rat’s serum calcium and total proteins levels after 1 and 2 months of treatment by AC and AZ doses

Level (mg/dL)		Acetamiprid doses			Azoxystrobin doses			Control
Parameters	Duration	1/10 LD_50_	1/20 LD_50_	1/40 LD_50_	1/10 LD_50_	1/20 LD_50_	1/40 LD_50_	
Calcium	1st month	7.32 ± 0.13 ^a^	8 ± 0.15 ^a,b^	9.91 ± 0.18 ^a,b^	9.16 ± 0.15 ^a^	10.03 ± 0.15 ^a,b^	11 ± 0.18 ^a,b^	12.02 ± 0.06
	2nd month	8.54 ± 0.12 ^a^	9.18 ± 0.14 ^a,b^	11.09 ± 0.17 ^a,b^	10.36 ± 0.15 ^a^	11.09 ± 0.17 ^a,b^	12.20 ± 0.16 ^a,b^	13.29 ± 0.12
Total proteins	1st month	5.25 ± 0.09 ^a^	5.79 ± 0.11 ^a,b^	7.07 ± 0.08 ^a,b^	6.21 ± 0.14 ^a^	6.96 ± 0.12 ^a,b^	7.62 ± 0.07 ^a,b^	8.06 ± 0.03
	2nd month	6.75 ± 0.09 ^a^	7.33 ± 0.08 ^a,b^	8.57 ± 0.08 ^a,b^	7.71 ± 0.14 ^a^	8.46 ± 0.12 ^a,b^	9.12 ± 0.07 ^a,b^	9.58 ± 0.01

Data presented as mean ± S.E. ^a^ Significant difference (1/10, 1/20, 1/40) LD_50_ as compared to the control group, and ^b^ significant difference as compared to the 1/10 LD_50_ group (*n* = 4)

At lower dosages of AC and AZ 5 mg/kg/day, 125 mg/kg/day, serum protein was 8.57 mg/dL and 9.12 mg/dL, respectively. At high AC and AZ doses, TP levels were 6.75 mg/dl and 7.71 mg/dL, respectively, while the TP of control animals was 9.58 mg/dl after 2 months of the treatment (Table 1).

The serum SOD levels were shown to be substantially lower at low dosages of AC and AZ, at 60.33 mg/mL and 68 mg/mL, respectively (Table 2). The serum CAT levels were 42.67 mg/mL and 51.67 mg/mL, respectively, at low dosages of AC and AZ (Table 2). Increasing the doses of AC and AZ to 20 mg/kg/day and 500 mg/kg/day, CAT levels declined to 31.00 mg/mL and 38.67 mg/mL, respectively, as compared to control animals (59.5 mg/mL).

**Table 2:** Rat’s serum oxidative/antioxidant biomarkers levels after 1 and 2 months of treatment by AC and AZ doses

Oxidative/antioxidant parameters		Acetamiprid doses			Azoxystrobin doses			Control
Levels (mg/mL)	Duration	1/10 LD50	1/20 LD50	1/40 LD50	1/10 LD50	1/20 LD50	1/40 LD50	
Catalase	1^st^ month	35 ± 1.15 ^a^	41.33 ± 1.45 ^a,b^	46.67 ± 1.20 ^a,b^	42.67 ± 1.45 ^a^	42.67 ± 1.15 ^a,b^	52.33 ± 0.20 ^a,b^	55.5 ± 0.29
	2^nd^ month	31 ± 1.15 ^a^	37.33 ± 0.88 ^a,b^	42.67 ± 1.20 ^a,b^	38.67 ± 1.45 ^a^	45 ± 1.15 ^a,b^	51.67 ± 0.88 ^a,b^	59.5 ± 0.29
Superoxide dismutase	1^st^ month	46.67 ± 1.86^a^	55.67 ± 1.76 ^a,b^	65.33 ± 1.45 ^a,b^	58 ± 1.53 ^a^	68 ± 0.58 ^a,b^	70.40 ± 0.31 ^a,b^	74.67 ± 0.88
	2^nd^ month	41.67 ± 1.86 ^a^	49 ± 0.88 ^a,b^	60.33 ± 1.45 ^a,b^	53 ± 1.52 ^a^	63 ± 0.8 ^a,b^	68 ± 1.15 ^a^	77.5 ± 0.29
Glutathione reductase	1^st^ month	28.67 ± 0.88 ^a^	35.33 ± 1.45 ^a,b^	40.8o ± 0.42 ^a,b^	40 ± 0.58 ^a^	43.50 ± 0.29 ^a,b^	45.27 ± 0.37 ^a,b^	48.5 ± 0.29
	2^nd^ month	23.67 ± 0.88 ^a^	30.33 ± 1.45 ^a,b^	36.33 ± 0.88 ^a,b^	35.67 ± 1.20 ^a^	40 ± 0.58 ^a,b^	43 ± 0.67 ^a,b^	55.5 ± 0.29
Nitric oxide	1^st^ month	72.33 ± 0.58 ^a^	64.33 ± 0.88 ^a,b^	57.67 ± 0.95 ^a,b^	57 ± 0.88 ^a^	45.67 ± 0.87 ^a,b^	40.33 ± 0.88 ^a,b^	33 ± 0.58
	2^nd^ month	76.33 ± 0.88 ^a^	68.33 ± 0.88 ^a,b^	61.67 ± 0.88 ^a,b^	61.67 ± 0.88 ^a^	49.67 ± 0.88 ^a,b^	44 ± 0.58 ^a,b^	36.9 ± 0.08

Data presented as mean ± S.E (*n* = 4). ^a^ Significant difference of 1/10, 1/20, and 1/40 LD_50_ as compared to the control group, ^b^ significant difference as compared to the 1/10 LD_50_ group

As seen in Table 2, rat serum GR was found to be lower in all doses when compared with the control group. After 2 months, AC (5 mg/kg/day) and AZ (125 mg/kg/day) changed the GR level to 36.33 and 43.67 mg/mL, respectively. In comparison to control rats, the AC and AZ caused a substantial rise in serum NO at all doses and during the study (Table 2).

### Fertility disorders

**Fig. 1:**
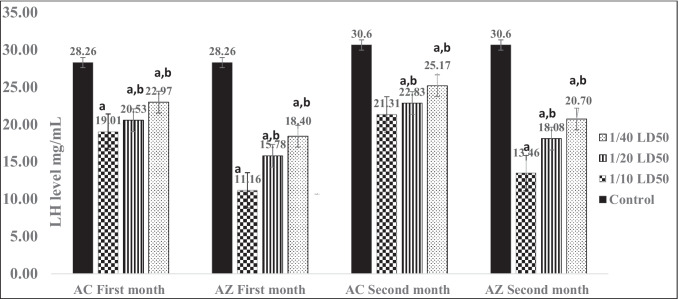
Rat’s serum LH levels after 1 and 2 months of treatment by AC and AZ doses. Data presented as mean ± S.E. ^a^Significant difference (1/10, 1/20, 1/40) LD_50_ as compared to the control group, ^b^ significant difference as compared to the 1/10 LD_50_ group (*n* = 4)

**Fig. 2:**
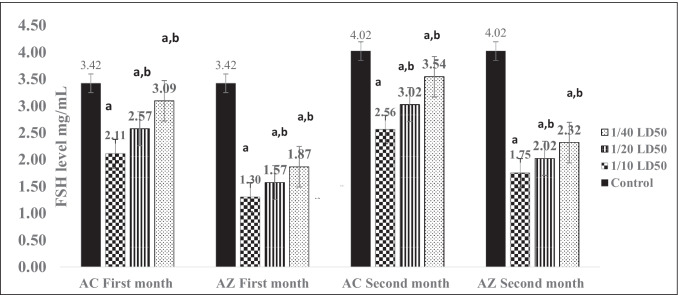
Rat’s serum FSH levels after 1 and 2 months of treatment by AC and AZ doses. Data presented as mean ± S.E. ^a^ significant difference (1/10, 1/20, 1/40) LD_50_ as compared to the control group, ^b^ significant difference as compared to the 1/10 LD_50_ group (*n* = 4)

When comparing the LH and FSH levels after AC and AZ treatment to the control rats, the LH levels were significantly lower at both doses (Fig. 1). At low doses of AC and AZ (5 and 125 mg/kg/day, respectively), LH levels were 20.7 and 25.17 mg/mL. The LH values were 13.46 and 21.31 mg/mL at high doses (20 and 500 mg/kg/day), respectively, as a consequence of AC and AZ, with a control group of 30.6 mg/mL. Rat FSH values at low doses of AC and AZ, on the other hand, were 2.32 and 3.54 ng/mL, respectively (Fig. 2). At massive doses of AC and AZ (20 and 500 mg/kg/day, respectively), FSH levels were 1.75 ng/mL and 2.56 ng/mL, respectively, while the control group's FSH was 4.02 ng/mL (Fig. 2).

### Sperm count and motility

After 1 and 2 months of treatment, the rat sperm count was slightly lower in AC and AZ treatments. In addition, the second-month findings changed significantly from the first-month results (Table 3). However, 1/10, 1/20, and 1/40 LD_50_ AC doses resulted in sperm counts of 84, 90.33, and 96.67 million/mL, respectively, compared to a control rat count of 111 million/mL.

**Table 3:** Rat’s sperm count and motility after 1 and 2 months of treatment by AC and AZ doses

Semen evaluation		Acetamiprid doses			Azoxystrobin doses			Control
Sperm	Duration	1/10 LD_50_	1/20 LD_50_	1/40 LD_50_	1/10 LD_50_	1/20 LD_50_	1/40 LD_50_	
Count (million/mL)	1^st^ month	84 ± 1.15^a^	90.33 ± 0.88 ^a,b^	96.67 ± 0.88 ^a,b^	93.33 ± 0.88 ^a^	98 ± 1.15 ^a,b^	101.67 ± 1.45 ^a,b^	110.67 ± 1.76
	2^nd^ month	87.33 ± 0.67 ^a^	93.67 ± 0.88 ^a,b^	98.33 ± 1.20 ^a,b^	91 ± 1.0 ^a^	99 ± 0.58 ^a,b^	106 ± 1.15 ^a,b^	120 ± 1.15
Motility (%)	1^st^ month	73.33 ± 0.88 ^a^	76.33 ± 0.88 ^a,b^	79 ± 0.58 ^a,b^	80.67 ± 0.67 ^a^	87 ± 1.0 ^a,b^	92.33 ± 0.88 ^a,b^	90.33 ± 0.88
	2^nd^ month	68 ± 1.15 ^a^	73 ± 1.73 ^a,b^	78.67 ± 0.88 ^a,b^	76 ± 1.15 ^a^	82.33 ± 1.45 ^a,b^	87.67 ± 1.20 ^a,b^	90.67 ± 1.20

Data presented as mean ± S.E. ^a^ significant difference (1/10, 1/20, 1/40) LD_50_ as compared to the control group, ^b^ significant difference as compared to the 1/10 LD_50_ group (*n* = 8/group; *n* = 4 at each time)

However, the sperm counts after 1/10, 1/20, and 1/40 LD_50_ AZ doses were 93.33, 98, and 101.67 million/mL, respectively, in the first month. The sperm count was 87.33, 93.67, and 98.33 million/mL, respectively, after separate doses of 1/10, 1/20, and 1/40 LD_50_ AC, compared to a control animal count of 120 million/mL. The sperm count at the end of the second month was 91, 99, and 106 million/mL, respectively, as a result of 1/10, 1/20, and 1/40 LD_50_ AZ (Table 3).

After 1 and 2 months of treatment, the motility of rat sperm was dramatically reduced as the concentration of the two pesticides increased relative to the control group (Table 3). In addition, the second-month findings differed significantly from the first-month results. In the first month, sperm motility was 73.33%, 76.33%, and 79%, respectively, as a result of 1/10, 1/20, and 1/40 LD_50_ AC, with a control rat count of 90%. After a month, the sperm motility percentages as a result of 1/10, 1/20, and 1/40 LD_50_ AZ doses were 80.67%, 87%, and 92.33%, respectively. In comparison to a control sample, the second-month results after separate doses of 1/10, 1/20, and 1/40 LD_50_ AC were 68%, 73%, and 78.67%, respectively. In comparison to control rats, sperm motility in the second month was 76%, 82.33%, and 87.67% as a result of 1/10, 1/20, and 1/40 LD_50_ AZ doses, respectively (Table 3).

### Histopathological observation

**Fig. 3:**
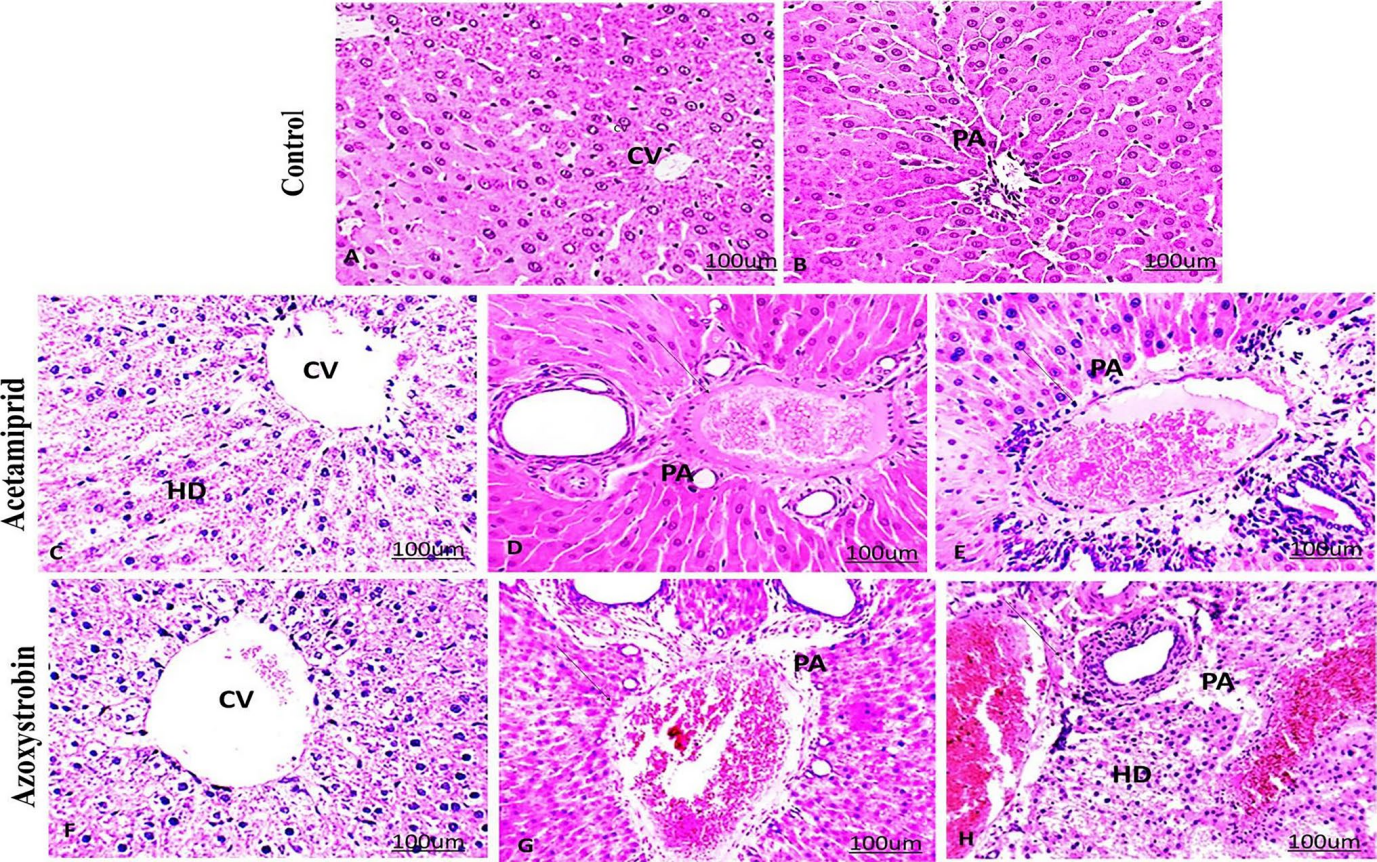
Effect of acetamiprid and azoxystrobin on liver tissue of rats. **A**, **B** Histological sections of the liver of control rats showed normal liver central vein (CV) and a normal portal area (PA). Liver sections of the treated group with acetamiprid with different doses (**C**–**E**). **C** Low dose demonstrated hydropic degeneration (HD), **D** medium dose demonstrated infiltration of mononuclear cells (arrow), hypertrophied and edema of portal area (PA), **E** high dose demonstrated infiltration of mononuclear cells (arrow), hypertrophied and edema of portal area (PA). Liver sections of the treated group with azoxystrobin (F–H). F; low dose demonstrated hydropic degeneration (HD). **G** Medium dose demonstrated infiltration of mononuclear cells (arrow), hypertrophied, and edema of portal area (PA). **H** High dose demonstrated infiltration of mononuclear cells (arrow), hypertrophied, and edema of portal area (PA). (hematoxylin–eosin (H&E) stained, × 200)

**Fig. 4:**
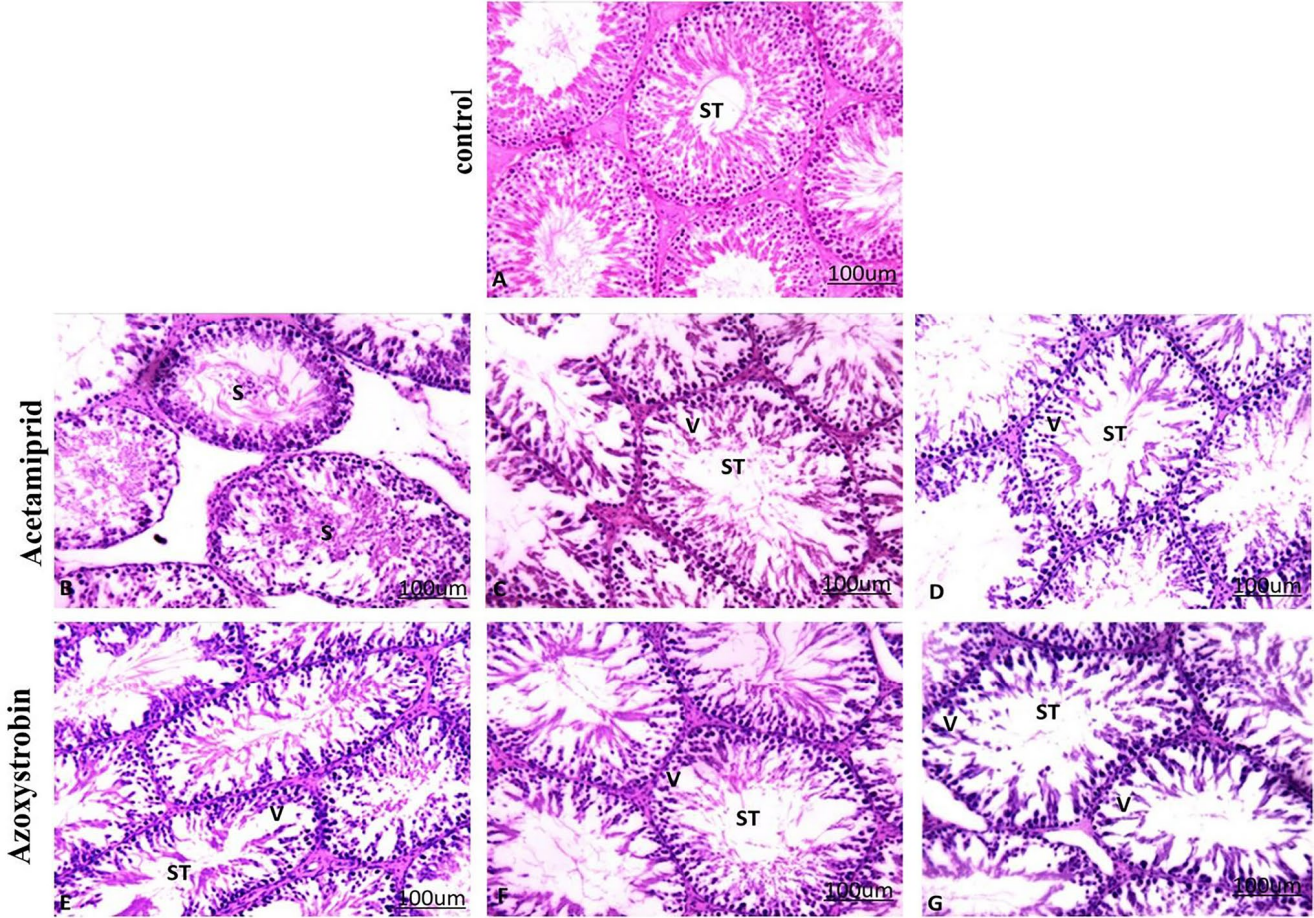
Effect of acetamiprid and azoxystrobin on testis tissue of rats. **A** Seminiferous tubules (ST) in the control group showed a normal appearance. **A** photomicrograph of seminiferous tubules (ST) of the treated group with acetamiprid. **B** Low dose demonstrated sloughing of germinal epithelium (S). **C** Medium dose demonstrated degenerated spermatogenic cells with vacuole formation (V). **D** High dose demonstrated degenerated spermatogenic cells with vacuole formation (V). Seminiferous tubules (ST) of the treated group with Azoxystrobin. **E** Low dose demonstrated degenerated spermatogenic cells with vacuole formation (V). **F** Medium dose demonstrated degenerated spermatogenic cells with vacuole formation (V). **G** High dose demonstrated degenerated spermatogenic cells with vacuole formation (V). (hematoxylin–eosin (H&E) stained, × 200)

The histopathological results of the liver and testes for each group of treatment of AC and AZ are represented in Figs. 3 and 4, respectively. There were no variations in the histopathological severity between the groups’ treatments.

The livers of the control group showed no histopathological alterations. The control liver tissue was histologically examined and revealed normal large hexagonal hepatocytes organized into cords radiating around the central vein and portal region. Blood sinusoids separate each cord of hepatocytes from the others (Fig. 3A and B).

Histopathological variations in the liver of AC (Fig. 3C–E) and AZ-treated classes (Fig. 3F–H) displayed portal mononuclear cell invasion, hydropic degeneration, and congestion of the central vein and hepatic artery, as well as veins that seemed dilated. Following AZ treatment, the liver undergoes significant histopathological changes. In the low-and high-dose groups of AC and AZ, edema and eosinophilia were found in the tissue (Fig. 3G–H).

The control group’s testis had a normal testicular histological structure and usual spermatogenesis. The ordered development from spermatogonia to spermatocytes with groups of spermatids and mature spermatozoa is seen in the seminiferous tubules with the usual thin basement membrane as well as the standard germinal epithelium (Fig. 4A). The number of spermatogenic cells has decreased with vacuole shape, spermatids, and certain cells sloughed into the lumen of the seminiferous tubules at all doses of AC (Fig. 4B–D) or AZ (Fig. 4E–G).

**Fig. 5:**
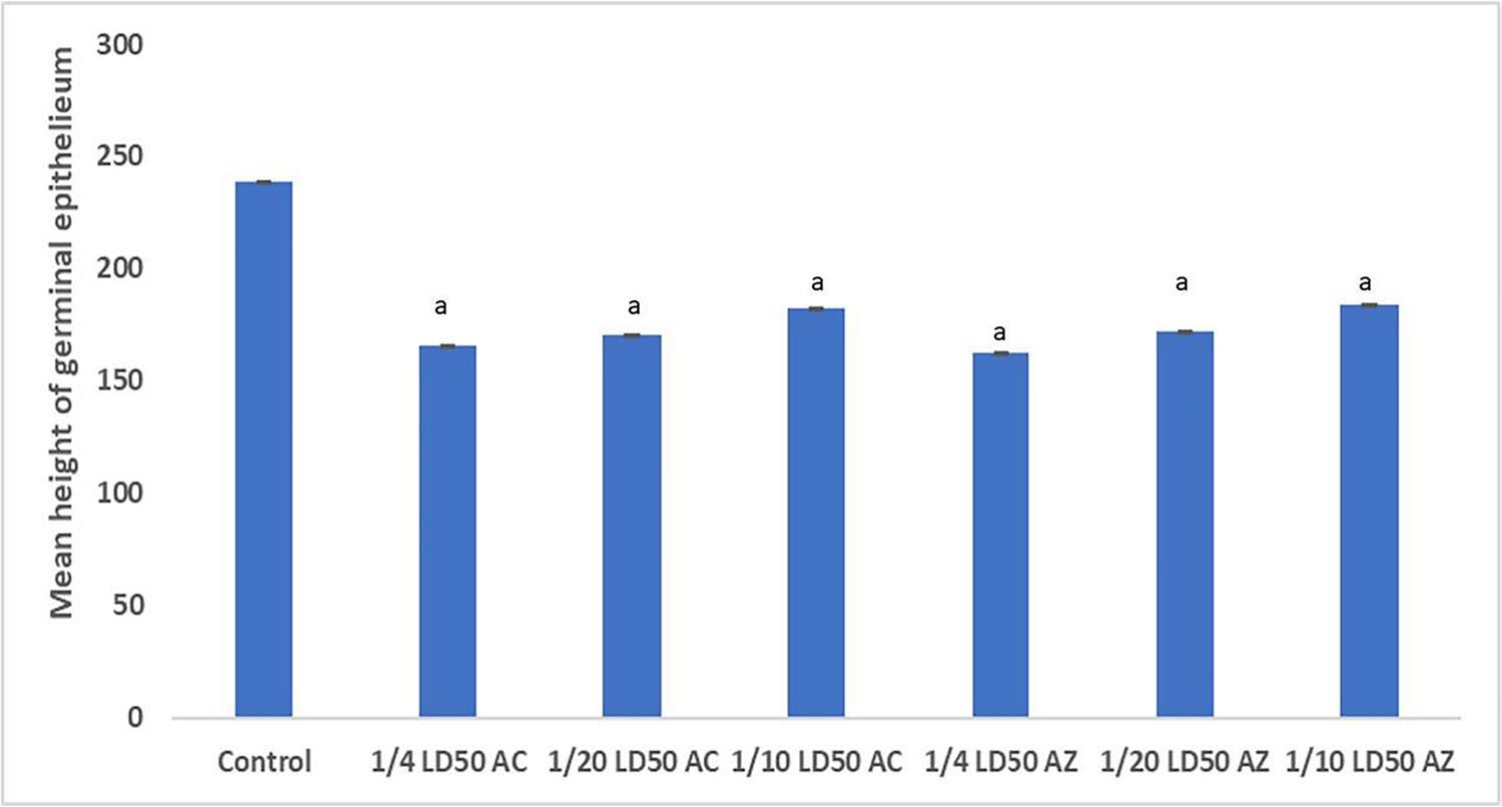
Effect of different doses of acetamiprid and azoxystrobin on the germinal epithelium. ^a^ Significant difference (1/10, 1/20, 1/40) LD_50_ as compared to the control group

The effect of different doses of acetamiprid and azoxystrobin on the germinal epithelium has been measured to prove the decrease in the spermatogenic cells (Fig. 5).

There was a significnant differences in germinal tissue in all treated groups compared to the control.

## Discussion

After a month and 2 months of AC and AZ treatment, the serum Ca, SOD, CAT, GR, NO, LH, and FSH levels, as well as histopathological alterations in the liver and testis were assessed. The effect of AC and AZ were estimated on sperm counting and their motility.

The rat serum Ca was impaired by AC and AZ, and at elevated doses, AC and AZ caused a noticeable deviation from the control group. The Ca levels were greatly reduced at 20 and 500 mg/kg/day of AC and AZ, respectively. This finding has previously been confirmed, while the equilibrium of bone formation and Ca homeostasis is influenced by both endogenous and exogenous influences (Ronis et al. 2020) Also, etoxazole, an oxazoline pesticide, induced a deficiency in Ca homeostasis (Ham et al. 2020).

During uremia, calcium synthesis is disrupted, which contributes to the progression of secondary hyperparathyroidism of the kidneys in monogastric patients (Kaneko and Tomino 1999). Analytically imperceptible hypocalcemia was observed in renal dysfunction, which may be a direct physiochemical consequence of hyperphosphatemia, a lack of active sources of vitamin D, or both. Hypocalcemia causes the release of parathyroid hormone, which raises the blood calcium level (Mondal et al. 2019).

In the current study, TP levels declined in a dose-dependent manner. This result was matched with previous observations. For example, the serum TP of male rats significantly decreased when exposed to phthalate esters (Di Lorenzo et al. 2020); additionally, the serum TP of female rats was considerably lower when exposed to biphenyl amine (Moustafa and Ahmed 2016; Di Lorenzo et al. 2020).

In the current findings, protein loss could be due to structural protein lysis, which is visible histologically as hepatocellular membrane disruption. These data are in coincidence with Bhushan et al. (2013) who found that cypermethrin and beta-cyfluthrin depleted TP levels and caused hepatocellular damage. Also, it may occur due to a disturbance in protein metabolism as a result of a reduction in protein synthesis or elevation of proteolytic activity or degradation (Uboh et al., 2009). Furthermore, the negative effects of ROS created by AC exposure on proteins oxidized in the liver may cause the TP drop. Although neonicotinoid pesticides have a variety of modes of action, they induce oxidative stress by inhibiting antioxidants and causing the formation of ROS (Çavaş et al., 2014).

The cellular enzymatic antioxidant protection mechanism defends biological structures from ROS by controlling the formation of free radicals and their metabolites in normal cells (Patlevič et al. 2016). The most effective antioxidant enzymes in the struggle against superoxide radicals are SOD, CAT, and GR. The dismutation of superoxide into oxygen and H_2_O_2_ is catalyzed by SOD (Patlevič et al. 2016). CAT is a heme-containing peroxisomal enzyme that plays a role in the breakdown of intracellular H_2_O_2_. The GR is another important antioxidant enzyme that uses NADPH to convert glutathione disulfide to sulfhydryl type glutathione. These enzymes work together in the ROS metabolic pathway to eliminate the oxidative stress effects (Rives et al. 2020).

The AC generated oxidative damage and impaired the liver, altering cholesterol levels, alanine aminotransferase, and aspartate aminotransferase enzymes (Karaca et al. 2019). Furthermore, at doses of 26.25 and 105 mg/kg for 28 days in Wistar rats, it caused LPO and diminished GSH production in the kidney and liver (Doltade et al. 2019). In the livers of rats exposed to higher doses of AC, Chakroun et al. (2016) found a statistically significant decline in SOD and CAT, as well as an increase in LPO. After sub-acute exposure to AC, similar findings were obtained in the testis of mice (Zhang et al. 2011).

Previous research showed a sliver of data and demonstrated the mechanism of EDC-induced induction of toxic effects. Testicular oxidative stress, for example, has been linked to pathological disorders including cryptorchidism, varicocele, and testicular torsion, as well as intoxicant exposures that are believed to damage male fertility (Turner and Lysiak 2008). Furthermore, phthalate inhibits the activity of enzymes involved in the synthesis of H_2_O_2_, resulting in changes in the activity of enzymes involved in the degradation of H_2_O_2_ (Arteel 2003).

Previous research suggested that AC’s effects occurred by elevation of oxidative stress, as it raised LPO and NO levels in the testes and decreased CAT activity (Mondal et al., 2019). In the same line as the previous explanation, AC and AZ increase NO levels and cause depletion of SOD, CAT, and GR at different doses. This change may be the result of the body's defensive mechanisms attempting to deal with the issue, resulting in a disruption of most biological functions and parameters. During the previous biochemical studies, there were significant changes in male rats' serum overall antioxidant levels during cypermethrin treatments (Bhushan et al., 2013). Biphenyl amine also caused significant alterations in antioxidant enzyme levels (Moustafa and Ahmed 2016). Moreover, exposure to AC resulted in a significant decrease in the levels of SOD and CAT activities with a concomitant increase in the LPO of the liver (Chakroun et al., 2016).

Our data show that AC treatment causes oxidative stress and increases serum NO levels. The antioxidant defense system's failure to eliminate ROS influx is evidenced by the overproduction of ROS and a decrease in SOD and CAT. The consumption of this enzyme in eliminating toxins may explain the reduced activity of SOD in the serum to remove O_2_^−^ into H_2_O_2_ while CAT, a ubiquitous enzyme, scavenges H_2_O_2_ into water and oxygen (Tang et al. 2006). However, the activity of these antioxidant enzymes involved in free radicals may be changed during these processes, resulting in a buildup of H_2_O_2_ in the cell membrane, which promotes LPO.

NO is a free radical produced by nitric oxide synthase (NOS) enzymes during catalytic oxidation. Long-term NO exposure reflects badly on tissue structure and integrity (Paul et al. 2016). This is due to the interaction of NO with O_2_ to produce reactive nitrogen oxide species like NO_2_ and N_2_O_3_, or with superoxide anion (O_2_) to produce highly reactive peroxynitrite (ONOO^−^), which can induce thiol oxidation or nitrosylation, tyrosine residue nitration, lipid, protein, and DNA oxidation (Abdel-Salam et al. 2017). The neurotoxic consequences of acute malathion poisoning in rats were related to increased endogenous NO production in the brain. In this study, we sought to see what role endogenously produced NO plays in the development of oxidative stress and neuronal damage caused by AC and AZ. The current findings strongly imply that excessive NO is produced by iNOS during AC and AZ intoxication.

Both dosages of AC and AZ significantly decreased LH and FSH levels when compared to healthy controls. This finding was in line with Moustafa and Ahmed's (2016) findings, which demonstrated that long-term exposure to biphenyl amine at dosages of 50 and 200 mg/kg BW induced significant alterations in sex hormones (LH and FSH). A reduction in serum LH levels was also seen owing to the disrupter action of nonylphenol (González-Fernández et al. 2008; Aly et al. 2012).

The results of the study show that AC or AZ treatment has a histopathological impact on the rat's liver and testis. The liver and testis of the treated groups showed a series of histopathological symptoms after exposure to AC or AZ. The liver was affected more than the testis, according to the histopathological results. Insecticide AC caused far more hepatocellular damage in rats than the fungicide AZ, which is mostly due to its systemic toxicity and metabolites. Our data coincides with the observations of Mesnage and Antoniou (2018). Previous research demonstrated that AC is found at a higher level in the liver compared to the kidneys or testes (Zhang et al. 2011). Similar to our histological findings, AC is more toxic to the liver compared to the testis. Histopathological evaluations of the liver showed a dose-dependent degenerative pattern.

Ziada and Abdulrhman (2020) reported that the AZ fungicide induced liver and kidney damage, as shown by the elevated function bioindicators. As a result of the AZ impact, GSH was reduced while MDA was increased dramatically. Aside from that, some tubular epithelial cells in the kidney were degenerating, there was hemorrhage, and the liver had an inflammatory cell infiltration.

As a result, the dose–response relationship was established in this study. The present study is complementary to Mondal et al. (2019) who reported that mild degenerative changes were observed in the liver of rats that received 25 mg/kg of AC, but the rats treated with 100 and 200 mg/kg showed severe liver fatty changes and necrosis, respectively. Also, in the present study, increased eosinophilia led to the lysis of hepatocytes.

The tissues showed hemorrhage, which seemed to be an allergic reaction to pesticides. The inflammatory response includes the migration and activation of both resident and circulating inflammatory cells, as well as the release of cytokines. Activated inflammatory cells and released cytokines induce fibroblasts to replicate, migrate, secrete and produce collagen (Ziada and Abdulrhman 2020).

Moreover, Mondal et al. (2019) reported that some chemicals may cause hepatocellular necrosis, rapid disorganization of the hepatic architecture, breakdown of sinusoidal structures, and pooling of blood in the liver through these mechanisms.

The testes of animals given AC or AZ show considerable degeneration of spermatogenic cells. Many researchers (Cao et al. 2019; Arıcan et al. 2020; Ibrahim et al. 2020) have recorded that AC or AZ affects the testicular histological structure, as well as spermatogenesis degeneration and sperm reduction. Also, Kong et al.’s (2017) study corresponds with the current data. Rats treated with AC (30 mg/kg BW for 35 days) showed that seminiferous tubules were impaired and had vacuolization as well as the number of interstitial Leydig cells decreased. In rat testes treated with AC (1/10 LD_50_/30 days), there was sloughing of spermatogenic cells, edema, a reduction in the number of sperm, and congestion (Keshta et al. 2016).

After 2 months of increasing the doses of AC and AZ, the rat sperm count and motility were dramatically reduced. This finding was previously recorded during a study of rats exposed to cyclophosphamide, which showed substantial differences in sperm count and morphology, as well as different effects on motility patterns (Shittu et al. 2019).

These biochemical elevations were confirmed by histopathological examinations as clear marked congestion, tubular cell degeneration, and sloughing of epithelial cells. Rats with reduced testosterone levels in their blood have less motile sperm after being exposed to AC (Halawa et al., 2021**)**. This effect may be caused by oxidative stress, which disrupts the lipid bilayer in the cell membrane, resulting in decreased testosterone biosynthesis, which causes sperm motility, viability, and dysfunctional sperm to decrease. Insecticides have also been indicated in previous studies to induce mitochondrial membrane impairment in Leydig cells and inhibit testosterone formation by reducing cholesterol transmission into the mitochondria and decreasing cholesterol transfer to pregnenolone in the cells, resulting in lower testosterone output. The AC-fed rats had fewer Leydig cells than usual diet-fed rats, which may explain why testosterone levels were reduced (Desai et al. 2017). Also, the nicotinic acetylcholine receptor (nAchRs) was activated by AC and suppressed gonadotropin activity, resulting in a hormonal imbalance that harmed sperm output (Ngoula et al., 2007).

In Sprague Dawley rats treated with 10 mg/kg and 30 mg/kg doses, Kong et al. (2017) found that AC raises LH levels while decreasing testosterone levels and sperm count. In Kunming male mice, Zhang et al. (2011) found that 30 mg/kg AC reduced the intact acrosome rate of sperm and testosterone levels. In a study performed by Mosbah et al. (2018), Wistar rats dosed with 27 mg/kg AC by gavage (5 days per week) for 45 days showed a rise in body weight and a decrease in testis weight, sperm count, plasma testosterone content, and sperm motility.

According to Arcan et al. (2020), oral administration of AC at varying doses (12.5, 25, and 35 mg/kg) for 90 days resulted in a dose-dependent drop in sperm concentration and plasma testosterone levels. Gonadotropin-releasing hormone (GnRH), FSH, and LH levels were all higher in the low and medium-dose cohorts. AC, on the other hand, caused LPO and GSH degradation in the testes. In both the low and high dose groups, the AC induced apoptosis, and the proliferation index in the high dose group was significantly decreased. Finally, AC toxicity in the reproductive system of males resulted from high doses. The oxidative stress, hormonal disruptions, and apoptosis could be the reasons for AC and AZ's toxic effects.

## Conclusion

Exposure to AC and AZ was found to induce liver and testes toxicity by increasing serum NO level and depletion of serum enzyme antioxidant activities (GSH, CAT, and SOD) and this was confirmed by histopathology of the liver and testes as well as the alteration in the hormonal level and sperm counting. Also, AC harmed sperm features, which influenced FSH and LH levels. The findings of this study might be beneficial in assessing the hazards of AC and AZ to reproductive health. As a result, because the effects of these pesticides are time and dose-dependent, their use should be restricted.

## Data Availability

All the data are presented in the paper.

## Declarations

### Data statement

The user data to support the findings of this study are available from the corresponding author upon request.

All authors have seen and approved the final version of the manuscript being submitted. They warrant that the article is the authors’ original work, has not received prior publication, and isn't under consideration for publication elsewhere.
